# Modern advances in disaster victim identification

**DOI:** 10.1080/20961790.2019.1678798

**Published:** 2019-12-19

**Authors:** Peter Ellis

**Affiliations:** Forensic Medicine and Pathology Consultancy, Adjunct Professor of Forensic Medicine and pathology, Griffith University, Brisbane, Australia; Adjunct Professor, School of Public Health and Social Services, Queensland University of Technology, Brisbane, Australia; Chair, Pathology and Anthropology Sub-Working Group of INTERPOL DVI Working Group

## Abstract

**Figure:** Video abstract Watch the video on Vimeo

Watch the video on Vimeo

It is a sad indictment of modern society that the mass media on which it relies for information about the current world has become almost immune to the impact of mass fatality. Major disasters become indistinguishable from the rest of the news, whether such incidents are of natural or unnatural causation. In this way the occasion of multiple deaths occurring in one incident becomes just another headline. Sadly, this means that the very real distress that is inevitably felt by the relatives and friends of victims of these incidents becomes forgotten in the ensuing chaos and upheaval that frequently follows such disasters.

Multi-fatality incidents vary dramatically in their scale, political and geographical situation as well as causation, but what is universal is the fact that every individual death means that relatives and loved ones have lost an important part of their lives. While different cultures and religions view death differently, most, albeit not all, are cognisant of the fact that loved ones need to grieve and that this grieving process is a very real part of normal life and permits surviving friends and family to continue their lives in a positive way despite the loss of someone special. The most important initiating factor in this grieving process is the confirmation and knowledge that the loved one has in fact died. Sadly, the confusion that frequently accompanies multi-fatality incidents, whether they be due to a large natural disaster, a significant transportation accident or even a criminal/terrorist incident, means that confirmation that particular individuals have in fact died may be either lacking or at least significantly delayed.

In many disaster plans initiated by public health and government authorities, detailed organisational planning terminates at the confirmation that death has occurred and the bodies are “sent to the mortuary”. There is, frequently, little understanding that identification of individual bodies and even bodily fragments (in the event of many major disasters) needs to be the first and perhaps most important process to be undertaken to initiate and support the grieving process that allows families and the whole community to react to these incidents in a manageable manner. This process has been given the label Disaster Victim Identification (DVI).

The identification of the dead has been an issue for forensic services for many years although it is only relatively recently that modern technological advances and major managerial procedures have been applied in an orderly and well-planned manner to this sometimes chaotic logistic problem. The concept of forensic identification has been actively progressing for many years and the development of modern fingerprint technology and the use of genetic profiling have been applied to individual forensic casework for some time, over a century in the case of ridgeology. However, the logistical confusion that is almost inevitable when a multi-fatality incident occurs means that the application of these scientific technologies may not be applied in the most efficient manner. Review of the identification processes conducted around the world reveals a number of different operational procedures. While the majority of medicolegal and police jurisdictions utilise the system advocated and managed by INTERPOL (The International Criminal Police Organization), there are other practices and procedures in use especially within North America and in parts of the Far East. Given that many multi-fatality incidents involve victims from countries around the world, it is inevitable that the investigators who are tasked with identifying these victims may also be trained, have gained experience and work under the jurisdictional control of many different countries. This can cause confusion especially when these investigative teams are tasked to work together. Therefore, it is appropriate that some uniformity in the identification process be introduced, especially when it involves multi-jurisdictional victims and investigators. This special issue of the Journal of *Forensic Sciences Research* does not purport to solve this multinational problem. Instead experienced and well-regarded investigators in several fields of forensic science have offered their modern take on the current state of DVI in their particular science.

The use of the digital capture of fingerprints in the setting of DVI is discussed by Johnson and Riemen [1] as this technology has revolutionised the whole science and application of ridgeology. They describe the development of fingerprint technology and demonstrate how the recent advances utilising digital fingerprint capture have dramatically improved both the accuracy and efficiency of the use of this methodology in the context of mass fatality investigation.

Sometimes considered the slowest and most expensive procedure to implement within the context of mass fatality investigation, genetic profiling is also the technology probably undergoing the most rapid and dramatic improvements and advancement over recent years. Tillmar et al. [2] have presented an impressive introduction to the use of massive parallel sequencing (MPS) in the context of the DNA identification of compromised biological samples. This is of particular importance as the very nature of mass fatality incidents is such that many of the remains are degraded and/or decomposed and obtaining good detailed DNA profiles from such samples may present significant challenges. This technology is still in its infancy but it is of immense interest that it can be introduced at this early stage so that positive comparative investigations can be effectively conducted.

The use of forensic odontology frequently plays a major role in identification after many multi-fatality incidents. As with all applied methods, it is dependent on the availability of adequate antemortem dental records together with the skill and methodology of the postmortem examination of the structures of the dentition and surrounding tissues and finally upon effective and accurate comparison of these antemortem and postmortem observations. In his comprehensive review, Forrest [3] has outlined not only the widespread current practice of forensic odontology in the context of DVI but also introduces some recent advances, many of which involve imaging technology, which are bringing this procedure to the forefront of efficient and accurate identification.

Many disasters result in the significant destruction and fragmentation of human remains thereby making examination and, especially, identification very challenging. The role of the forensic anthropologist continues to be enhanced by the presence of ever improving methodology together with the application of significant primary and applied research which allows this scientific approach to be particularly enhancing in the context of such an investigative process. In an extensive summary of the role of forensic anthropology in DVI, de Boer et al. [4] have illustrated the value of quality forensic anthropological expertise both at the scene of the disaster and in subsequent postmortem examination. This has been endorsed in the recent appendix on the use of forensic anthropology in DVI in the 2018 INTERPOL DVI Guide [5]. de Boer et al. [4] have also included the value of education and training in this specialty together with the advantage of including the identification of survivors in the context of many mass fatality incidents.

Finally, Barone and Di Maggio [6] have prepared an interesting review of ground penetrating radar (GPR), which is a technique not frequently considered in the aftermath of mass fatality incidents but which can be very useful when the investigation undertaken includes the exhumation of human remains, especially when those remains have been interred in locations that are not well documented. Such situations could include historical burials especially when identification of those interred remains is considered to be desirable or necessary. The location of such remains is a very obvious essential prerequisite to the long process that in identification.

As mentioned by de Boer et al. [4], while some of these technologies are reasonably considered to be accurate and reliable enough to be sufficient for identification in their own right (sometimes referred to as “primary identifiers”), it is reasonable to think that all the technologies should be taken into account in all situations and that the methodology in any incident should be on the basis of a multidisciplinary team approach given that every disaster is different and it may not be possible to determine early in the response period which of the methodologies will be more effective in producing the definitive identification result. It is encouraging to realise that research into many of these technologies continues around the world and is the subject of extensive investigation, discussion and application both through the various working groups of the INTERPOL DVI community as well as in those forensic environments in which other processes are used.

Peter Ellis *Forensic Medicine and Pathology Consultancy, Adjunct Professor
of Forensic Medicine and pathology, Griffith University, Brisbane,
Australia* *Adjunct Professor, School of Public Health and
Social Services, Queensland University of Technology, Brisbane,
Australia* *Chair, Pathology and Anthropology Sub-Working
Group of INTERPOL DVI Working
Group* forensicpath@bigpond.com

